# Influence of Neoadjuvant Therapy on Success of Endoscopic Vacuum Therapy in Anastomotic Leakage after Rectal Resection Because of Rectal Cancer

**DOI:** 10.3390/jcm13133982

**Published:** 2024-07-08

**Authors:** Rahel M. Strobel, Julia E. Wellner, Konrad Neumann, Susanne D. Otto, Sophie M. Eschlboeck, Claudia Seifarth, Christian H. W. Schineis, Katharina Beyer, Martin E. Kreis, Johannes C. Lauscher

**Affiliations:** 1 Department of General and Visceral Surgery, Charité—Universitätsmedizin Berlin, Corporate Member of Freie Universität Berlin and Humboldt-Universität Berlin, Campus Benjamin Franklin, Hindenburgdamm 30, 12203 Berlin, Germanyjohannes.lauscher@charite.de (J.C.L.); 2 Institute of Biometry and Clinical Epidemiology, Charité—Universitätsmedizin Berlin, Corporate Member of Freie Universität Berlin and Humboldt-Universität Berlin, Charitéplatz 1, 10117 Berlin, Germany; 3 Department of General and Visceral Surgery, St. Claraspital, Kleinriehenstrasse 30, 4058 Basel, Switzerland; 4 Executive Board, Charité—Universitätsmedizin Berlin, Corporate Member of Freie Universität Berlin and Humboldt-Universität Berlin, Charitéplatz 1, 10117 Berlin, Germany

**Keywords:** endoscopic vacuum therapy, anastomotic leakage, neoadjuvant radio-chemotherapy, rectal cancer

## Abstract

**Background:** For locally advanced rectal cancer, neoadjuvant therapy (NT) is an established element of therapy. Endoscopic vacuum therapy (EVT) has been a relevant treatment option for anastomotic leakage after rectal resection since 2008. The aim was to evaluate the influence of NT on the duration and success of EVT in anastomotic leakage after rectal resection for rectal cancer. **Methods:** This was a monocentric, retrospective cohort study including patients who underwent rectal resection with primary anastomosis because of histologically proven carcinoma of the rectum in the Department for General and Visceral Surgery of Charité—Universitätsmedizin Berlin, Campus Benjamin Franklin over a period of ten years (2012 to 2022). **Results:** Overall, 243 patients were included, of which 47 patients (19.3%) suffered from anastomotic leakage grade B with consecutive EVT. A total of 29 (61.7%) patients received NT and 18 patients (38.3%) did not. The median duration of EVT until the removal of the sponge did not differ between patients with and without NT: 24.0 days (95% CI 6.44–41.56) versus 20.0 days (95% CI 17.03–22.97); *p* = 0.273. The median duration from insertion of EVT until complete healing was 74.0 days with NT (95% CI 10.07–137.93) versus 62.0 days without NT (95% CI 45.99–78.01); *p* = 0.490. Treatment failure—including early persistence and late onset of recurrent anastomotic leakage—was evident in 27.6% of patients with NT versus 27.8% without NT; *p* = 0.989. Ostomy was reversed in 19 patients (79.2%) with NT compared to 11 patients (68.8%) without NT; *p* = 0.456. Overall, continuity was restored in 75% of patients in the long term after EVT. **Conclusion:** This trial comprised—to our knowledge—the largest study cohort to analyze the outcome of EVT in anastomotic leakage after rectal resection for rectal cancer. We conclude that neoadjuvant therapy neither prolongs EVT nor the time to healing from anastomotic leakage. The rates of treatment failure of EVT and permanent ostomy were not higher when neoadjuvant therapy was used.

## 1. Introduction

Colorectal cancer is the third most common cancer worldwide and the second leading cause of death in cancer patients [1]. In the multimodal treatment of rectal cancer, total mesorectal excision (TME) has become the standard procedure for curative oncological resection [2]. For locally advanced rectal cancer, neoadjuvant therapy (NT) is an established element of therapy. The aim is to downsize and downstage the tumor prior to surgery, which has been shown to decrease local recurrence rates and increase the rate of sphincter-saving surgery [3]. While preoperative short-course radiotherapy and radio-chemotherapy, both combined with postoperative chemotherapy, present comparable long-term results, total neoadjuvant treatment has gained increasing interest under the assumption of improved systemic control [4]. Rectal cancer treatment can lead to late toxicity, declined quality of life and compromised function, such as low anterior resection syndrome, incontinence or sexual dysfunction. Impairment is more often seen in patients who undergo NT and surgery compared to surgery alone [4].

Anastomotic leakage is a major complication in rectal cancer surgery and contributes to morbidity, mortality and a prolonged hospital stay, which creates distress in the patients and increases overall costs [5]. Furthermore, anastomotic leakage affects the long-term functional and oncological outcome and may lead to permanent ostomy, increased local recurrence rates and reduced cancer survival [6,7]. Reported rates of anastomotic leakage vary and are around 10–20% after low anterior resection [5,8,9]. 

Treatment options for rectal anastomotic leakage depend on the patient’s condition, grade and location of the defect and the presence of a diverting ostomy. While patients with general peritonitis still require re-laparotomy and resection of the anastomosis, stable patients with diverting ostomy can benefit from less invasive non-operative treatment, such as percutaneous image-guided drainage, endoscopic clip placement or endoscopic vacuum therapy (EVT) [10]. EVT was first described in 2008 and involves the endoscopic placement of a vacuum sponge into the insufficient cavity [11]. Continuous drainage enables granulation and a reduction in the cavity, thereby reducing bacterial contamination and secretion. As studies describe an 85–89% healing rate of leaks with consecutive preservation of the anastomosis [12,13], EVT has become an important treatment option in many institutions.

The aim of this monocentric, retrospective study was to evaluate the influence of neoadjuvant therapy on the duration and success of endoscopic vacuum therapy in anastomotic leakage after rectal resection for rectal cancer. In addition, we assessed the long-term outcome of EVT, such as re-operation, recreation of continuity and anastomotic stricture. The primary outcome was the duration of EVT from insertion until removal in days. Our hypothesis was that patients with NT had a longer duration of EVT in anastomotic leakage than patients without NT.

## 2. Material and Methods

### 2.1. Trial Oversight

This was a monocentric, retrospective cohort study comparing the duration and success of EVT in anastomotic leakage after rectal resection between patients with neoadjuvant therapy to patients without neoadjuvant treatment. Patients who underwent rectal resection with primary anastomosis because of histologically proven carcinoma of the rectum in the Department for General and Visceral Surgery of Charité—University Medicine Berlin, Campus Benjamin Franklin in the period between 1 October 2012 and 29 August 2022 were eligible.

### 2.2. Study Population

Patients who were 17 years or older and undergoing elective rectal resection were eligible to participate. The histologically proven carcinoma of the rectum had to be located ≤16 cm from the anocutaneous line. Different surgical techniques such as open, laparoscopically assisted or robotic assisted surgery were included. Patients undergoing anterior rectal resection as well as low anterior resection, intersphincteric resection and proctocolectomy with ileo-pouch-anal anastomosis were eligible. Patients undergoing emergency surgery or abdomino-perineal excision without creation of an anastomosis were excluded.

### 2.3. Anastomotic Leakage and Endoscopic Vacuum Therapy

The diagnosis of anastomotic leakage in our trial was confirmed by rectoscopy, by computed tomography with intravenous contrast agent or intraoperatively. In our study, anastomotic leakage was categorized according to grade A to C by Rahbari et al. Grade A was defined as a defect of the anastomotic site which results in no change in patient’s management. Grade B required interventions such as endoscopic treatment without re-laparotomy and in grade C, anastomotic leakage re-operation with re-laparotomy was needed [14].

In the case of anastomotic leakage with more than one-quarter of the circumference of the anastomosis, endoscopic vacuum therapy (EVT) was immediately applied to the patient by one of the surgeons. Patients with only a small dehiscence of the anastomosis were not treated with EVT, but with rinsing and regular endoscopic controls. We performed a flexible rectoscopy in our endoscopic unit under sedation or without, depending on the patient’s condition. We used an open-pored white foam for negative pressure wound therapy with the size 7.5 cm × 10 cm × 0.9 cm (Mondomed^®^, Hamont-Achel, Belgium, distributed by Smith & Nephew Medical Limited, Hull, UK) connected to a redon draining tube of 250 mL and 12 Charrière with a constant negative pressure. We cut down the sponge to fit in the insufficient cavity. The anastomosis was regularly examined by one of the surgeons—usually every three to four days—by flexible rectoscopy and the sponge was exchanged and downsized depending on the healing of the insufficient cavity. In case of unintentional loss of the sponge or a loss of negative pressure, the sponge change was carried out immediately. The examining surgeon determined the healing of anastomotic leakage. The sponge was removed if the anastomosis was intact, the cavity was closed by granulation and no necrosis or wound secretion was observed. The sponge was removed and transanal irrigation therapy with sodium chloride was started. Irrigation therapy was performed regularly if needed and was terminated when the complete healing of the anastomosis was achieved.

### 2.4. Outcomes

The primary endpoint was the duration of the endoscopic vacuum therapy in days until the removal of the sponge. The time between the insertion of the EVT and complete healing of the anastomotic leakage in days was recorded as well. Secondary outcomes were the number of changes in the sponge, operative revision, length of hospital stay in days and successful reversal of diverting ileostomy. Treatment failure of EVT was defined either as persistence of anastomotic leakage diagnosed during EVT or during transanal irrigation after removal of the sponge. This resulted in either resumption of EVT or in re-laparotomy with dissolving of the anastomosis via terminal colostomy. Failure of EVT was also defined as anastomotic leakage which became apparent after ileostomy reversal and led to recreation of ostomy. Patients’ characteristics and comorbidities as well as surgical details were recorded retrospectively.

### 2.5. Statistical Analysis

The primary outcome, duration of endoscopic vacuum therapy (EVT), was analyzed using Kaplan–Meier curves and Cox proportional hazard models. Metric variables with skewed distributions were assessed using the non-parametric Mann–Whitney U-test. Associations between categorical variables were examined through cross tabulation and Pearson’s chi-squared tests. Results from the Cox proportional hazard models were presented as odds ratios (ORs) with two-sided 95% confidence intervals and corresponding *p*-values. Categorical variables were described by providing relative and absolute frequencies, while quantitative parameters were summarized with medians, minimum and maximum values. Statistical analysis was performed using IBM SPSS Statistics 26^®^ (IBM, Armonk, NY, USA).

### 2.6. Ethics

The ethics committee of Charité—University Medicine Berlin (application number EA4/173/21 at 21 July 2021) approved the study. The trial was conducted in accordance with the ethical principles of the Declaration of Helsinki and the principles of Good Clinical Practice (ICH-GCP E6) [15].

## 3. Results

### 3.1. Baseline Characteristics

**Chart 1:**
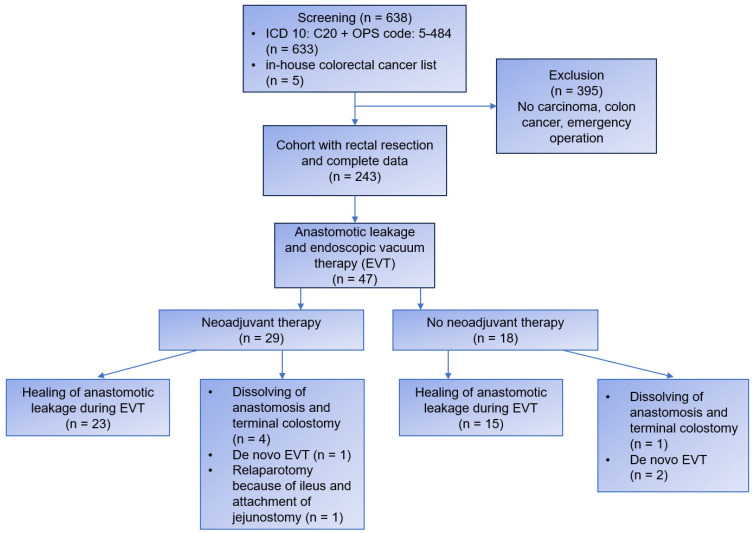
Patients overview.

Overall, 638 patients were screened for eligibility with the help of ICD 10 and OPS codes (see Chart 1). Overall, there were 243 patients who underwent rectal resection with primary anastomosis because of histologically proven carcinoma of the rectum that was located ≤16 cm from the anocutaneous line in the Department for General and Visceral Surgery of Charité—Universitätsmedizin Berlin, Campus Benjamin Franklin in the period between 1 October 2012 and 29 August 2022. The cohort of this study contained 47 patients who were treated with EVT. 

Patient characteristics of the cohort treated with EVT are summarized in Table 1. The cohort comprised ten (21.3%) females and 37 (78.7%) males. The overall median age was 63 years with a minimum of 37 and a maximum of 85 years. More than half (51.1%) of the tumors were located in the middle third of the rectum (between 6 and 12 cm from the anocutaneous line), 29.8% were present in the upper third (12 to 16 cm) and 19.1% in the lower third of the rectum (0 to 6 cm from the anocuteaneous line).

### 3.2. Neoadjuvant Therapy

Twenty-nine (61.7%) patients received neoadjuvant therapy including radio-chemotherapy or exclusively radiotherapy or chemotherapy compared to eighteen (38.3%) patients without neoadjuvant treatment. Table 2 depicts the different regimes of neoadjuvant treatment of patients with EVT. Nearly one-third (27.6%) received long-term radiotherapy with 50.4 gray combined with capecitabine and 20.7% of the patients were treated with long-term radiotherapy for six weeks exclusively. Information about the regime was missing in 10.3% of patients. One patient (3.4%) of twenty-nine patients interrupted neoadjuvant radio-chemotherapy.

### 3.3. Surgical Characteristics

The duration of surgery did not differ between patients with and without NT: 370 min versus 326 min median; *p* = 0.309. There was no difference in the technique of anastomosis. A circular stapler was used in 75.9% in patients with NT versus 77.8% in patients without NT; *p* = 0.880. No significant difference was seen regarding the site of anastomotic leakage (*p* = 0.177). The size of the cave of insufficiency was 80 mm median in patients with NT compared to 40 mm median in patients without NT measured in the flexible rectoscopy; *p* = 0.111 (see Table 3).

### 3.4. Anastomotic Leakage and Consecutive Endoscopic Vacuum Therapy

Overall, 47 (19.3%) of 243 patients suffered from anastomotic leakage grade B according to Nuh Rahbari and required EVT. A total of 29 (61.7%) patients received neoadjuvant therapy and 18 patients (38.3%) did not receive NT. Neoadjuvant treatment was not associated with the occurrence of anastomotic leakage (*p* = 0.257). The median time between the operation and the onset of anastomotic leakage was 8 days in total.

### 3.5. Duration of Endoscopic Vacuum Therapy

**Figure 1:**
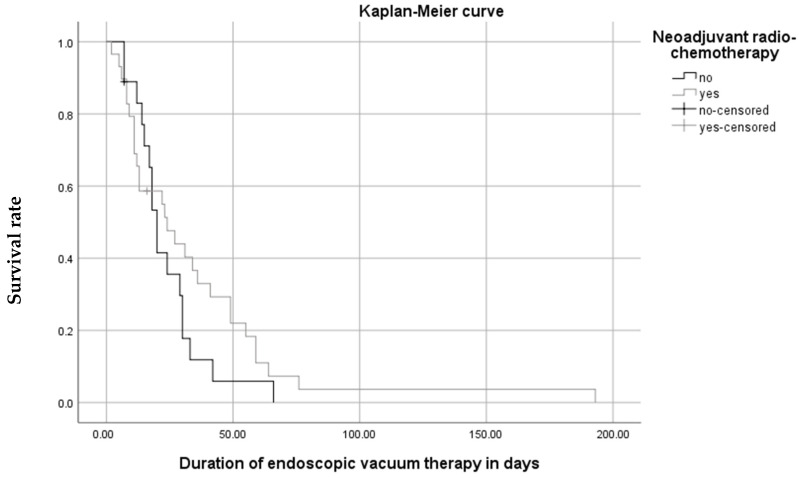
Kaplan–Meier curve of duration of endoscopic vacuum therapy until removal of sponge with or without neoadjuvant therapy.

The duration of EVT until the removal of the sponge did not differ between patients with and without neoadjuvant therapy (see Figure 1). The median duration of EVT was 24.0 days with NT (95% CI 6.44–41.56) versus 20.0 days without NT (95% CI 17.03–22.97); Log Rank: *p* = 0.273.

**Figure 2:**
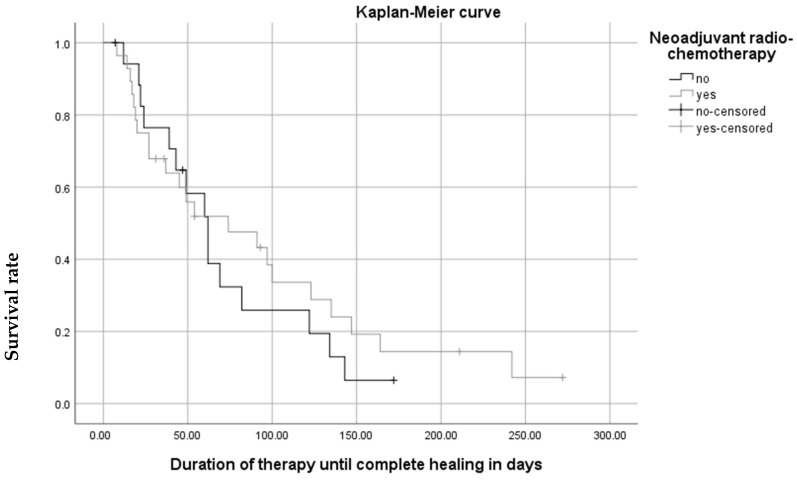
Kaplan–Meier curve of duration of therapy until complete healing of anastomosis with or without neoadjuvant therapy.

After the removal of the sponge, all the patients received consecutive clinical examinations with flexible rectoscopy and transanal irrigation with sodium chloride. The comparison of the duration of EVT until complete healing—end of transanal irrigation—between the two groups showed no difference (see Figure 2). The median duration from insertion of endoscopic vacuum therapy until complete healing was 74.0 days with NT (95% CI 10.07–137.93) versus 62.0 days without NT (95% CI 45.99–78.01); Log Rank: *p* = 0.490.

In Cox regression, there was no association between the duration of EVT and the variables neoadjuvant therapy (OR 1.18; 95% CI 0.56–2.45; *p* = 0.668), sex (OR 1.94; 95% CI 0.82–4.61; *p* = 0.132), age at operation (OR 0.99; 95% CI 0.96–1.02; *p* = 0.465), BMI (OR 1.13; 95% CI 0.99–1.27; *p* = 0.062), preoperative anemia (OR 0.97; 95% CI 0.46–2.06; *p* = 0.942) or ASA score (OR 0.87; 95% CI 0.41–1.82; *p* = 0.702).

### 3.6. Long-Term Outcome of EVT

The retrospective follow-up time after rectal resection varied between 16 and 126 months. No patient died during the hospital stay. Four patients died after initial discharge from hospital. Postoperative, anastomotic stricture occurred in four (13.8%) patients with NT compared to five (27.8%) patients without NT; *p* = 0.236 (see Table 4). Six anastomotic strictures were treated with transanal dilatation, one dissolving of the anastomosis was required, one stricture was incised and widened and one did not need operative treatment.

### 3.7. Recreation of Continuity

There were data about the reversal of protective ileostomy in fourty patients; information was missing in six patients and one patient did not have a protective ileostomy. Reversal of ostomy was performed in 19 patients (79.2%) with NT compared to 11 patients (68.8%) without NT; *p* = 0.456. Three patients overall had terminal ostomy after dissolving of the anastomosis because of persistent anastomotic leakage and underwent restoration of continuity. Overall, 75% of patients had restored continuity without ostomy. Overall, four (8.5%) patients suffered from late onset of recurrent rectal anastomotic leakage after initial EVT. All patients required recreation of ostomy—in three patients ileostomy reversal failed and an ileostomy was recreated, and in one patient re-laparotomy with dissolving of anastomosis and creation of terminal colostomy was performed.

### 3.8. Failure of Endoscopic Vacuum Therapy

Overall, persistence of anastomotic leakage during EVT occurred in nine patients (19.1%). There was no difference between patients with or without NT: six (20.7%) patients versus three (16.7%) patients; *p* = 0.733. Four patients (13.8%) with NT required dissolving of the anastomosis and creation of terminal colostomy compared to one patient (5.6%) without NT; *p* = 0.343. One patient with NT (3.4%) required re-laparotomy and creation of jejunostomy because of ileus. This patient did not suffer from anastomotic leakage and therefore anastomosis was not dissolved. EVT was applied de novo in one patient (3.4%) with NT and in two patients (11.1%) without NT; *p* = 0.134. The mean duration of de novo EVT was 16 days. Long-term treatment failure—including persistence of anastomotic leakage during EVT and late onset of recurrent anastomotic leakage after reversal of ostomy—occurred in 13 (27.7%) patients overall. There was no difference between patients with and without NT: 8 (27.6%) versus 5 (27.8%); *p* = 0.989 (see Table 4). The length of hospital stay was 24 (9–51) days median when neoadjuvant treatment was conducted and 25 (7–67) days median without it; *p* = 0.606.

## 4. Discussion

This monocentric, retrospective cohort trial comprised the largest population to assess the influence of neoadjuvant therapy on the duration and success of EVT in anastomotic leakage following rectal resection because of rectal cancer. The main finding is that the duration of EVT did not differ between patients with or without neoadjuvant therapy: 24 days versus 20 days. Neither the time between the insertion of the EVT and the complete healing of the anastomotic leakage nor the number of sponge changes were longer or higher in patients with NT than in patients without NT. There was no significant association between the duration of EVT and the variables sex, age at operation, BMI, preoperative anemia or ASA score. In our retrospective trial, persistence of anastomotic leakage during EVT that required re-operation or de novo EVT occurred in 19.1% overall. One factor explaining the high rate of anastomotic leakage was a rate of 83% of deep anterior and intersphincteric rectal resection bearing a higher risk for leakage than anterior resection. Furthermore, we often performed postoperative rectoscopy routinely before discharge, which contributed to early recognition of anastomotic leakage. Persistence of anastomotic leakage was not associated with the application of NT.

The duration of EVT was comparable with other studies [16,17,18]. A retrospective trial with 15 patients also found no longer duration of EVT in patients with neoadjuvant treatment [19]. Two trials—one prospective with 26 patients and one retrospective trial with 19 patients—depicted a longer duration of EVT in patients with NT [20,21]. Notably, one-third of patients without NT suffered from sigmoid cancer which has a different anatomy than the rectum. Another limitation in the trial of Bernstorff et al. is that patients with radio-chemotherapy had significantly lower anastomoses than patients without NT favoring lower healing time.

In our retrospective trial, the persistence of anastomotic leakage during EVT occurred in 19.1% overall. The persistence of anastomotic leakage was not associated with the application of NT. A multicenter trial from France also depicted that neoadjuvant radio-chemotherapy was not associated with the success of EVT [22]. In our trial, re-laparotomy and dissolving of the anastomosis with creation of terminal colostomy was necessary in five patients because of the persistence of anastomotic leakage during EVT and three patients required de novo EVT overall. Thus, endoscopic follow-up of the anastomosis is crucial after finishing EVT and the removal of sponge. Treatment failure—including early persistence of anastomotic leakage during EVT and late onset of recurrence of anastomotic leakage in our study—was evident in 27.7%. Riss et al. found a comparable rate of late treatment failure of 25%—defined as recurrent abscesses with a median time of 255 days after the end of EVT [16]. An even higher rate of 44.7% treatment failure after one year was shown by Abdalla et al. [22]. Treatment failure for EVT was lower in other trials between 5.6% and 14.7% [10,13,17,23]—keeping in mind that the definition varies in the literature. The existing trials did not explicitly assess the effect of NT on treatment failure. In our trial, the rate of treatment failure was not higher in patients with neoadjuvant treatment.

We could not find patient-related risk factors for the persistence of anastomotic leakage during EVT. In particular, neoadjuvant therapy had no risk factor in our study. Riss et al. also showed that neoadjuvant radio-chemotherapy had no significant influence on the occurrence of recurrent abscess after anastomotic leakage [16]. On the other hand, Shalaby et al. significantly associated preoperative radiotherapy, absence of protective ileostomy and male sex with treatment failure of EVT [13].

An expert panel consensus on the use of EVT for treatment of colorectal anastomotic leaks called for a standard definition for the success of EVT. They proposed to differentiate between technical (radiological and/or endoscopic examinations), clinical (restoration of intestinal tract) and long-term outcomes (satisfactory functional outcome and intestinal continuity after two years) [24]. In our trial, we used flexible rectoscopy as an endoscopic examination to determine whether the anastomotic leakage healed and EVT could be terminated. The decision made by the examining surgeon was based on the extent of granulation and closure of the cavity as well as the presence of necrosis or wound secretion. In our trial, 45 of 47 patients received transanal flush therapy with sodium chloride when the sponge was removed. After removal of the sponge, all the patients received consecutive clinical examinations—most of them in an outpatient setting—including flexible rectoscopy.

We think that our findings are important for patient-centered communication and for delivering adequate information about the expectable duration of EVT. We also assessed the long-term rate of restoration of continuity after EVT. Restoration of continuity after EVT was performed in 75% overall. The rate was comparable with other trials—rating between 63.8% and 75.9% [13,22,25]. Mahendran et al. depicted a lower rate of recreation of continuity of 51% than in our trial [10]. In our study, four (8.5%) patients suffered from the late onset of recurrent anastomotic leakage after initial EVT and required recreation of ostomy. We want to highlight the importance of a precise examination of the anastomotic site by digital rectal examination, flexible or rigid rectoscopy and rectal contrast enema before reversal of ileostomy is performed. Neoadjuvant treatment did not affect the rate of recreation of continuity in our trial.

The rate of anastomotic stricture after conservative treatment of colorectal or coloanal anastomotic leakage varies in the literature between 3% and 30% [22,26,27]. In our study, postoperative anastomotic strictures occurred in nine (19.1%) patients. Thereof, six patients were treated with transanal dilatation (66.7%), one dissolving of the anastomosis was required, one stricture was incised and widened and one did not need operative treatment. Neoadjuvant treatment was not associated with anastomotic stricture. Mussetto et al. depicted a comparable rate of 18.1% anastomotic strictures after EVT for anastomotic leakage during a mean follow-up of 29 months [23]. Abdalla found a lower rate of 10% of strictures that required instrumental dilatation [22].

We could assume that neoadjuvant therapy neither prolongs EVT nor the time to healing from the anastomotic leakage. This finding is important as it weakens the presumed negative impact of radio-chemotherapy on anastomotic healing.

Several potential limitations of the study need to be considered. First, this was a monocentric, retrospective study with inherent limitations. There was missing data or follow-up was incomplete. Still, data were complete in 93% of patients and follow-up was complete in 85% of patients, which is satisfactory for a retrospective study. The number of examined patients is quite low. Nevertheless, to our knowledge, this study represents the largest cohort to be examined regarding this research question. Second, there was inhomogeneity in the regimes of neoadjuvant therapy. Third, although there were standard operating procedures for EVT, EVT and its termination still differed slightly depending on the surgeon’s discretion. Fourth, there was a possibility for a type 2 error because of an underpowering of the trial. The cohort of 47 patients is small, thus a false negative result should be considered.

## 5. Conclusions

This trial comprised—to our knowledge—the largest published cohort which received EVT in anastomotic leakage following rectal resection for rectal cancer. We conclude that neoadjuvant therapy neither prolongs EVT, the time to healing from anastomotic leakage, the rate of treatment failure nor the rate of ostomy carriers. According to our study, neoadjuvant treatment in rectal cancer does not appear to have a negative impact on the healing of anastomotic leaks with endoscopic vacuum therapy.

## Figures and Tables

**Table 1:** Baseline characteristics.

	NT (*n* = 29)	No NT (*n* = 18)	Total (*n* = 47)	*p*-Value
Sex				0.391 §
Female	5 (17.2%)	5 (27.8%)	10 (21.3%)	
Male	24 (82.8%)	13 (72.2%)	37 (78.7%)	
Age (years)	62.0 (50.5, 69.5)	64.0 (57.3, 76)	63.0 (53, 71)	0.193 ^†^
BMI (kg/m^2^) Missing data = 1	25.6 (24.2, 29.3)	26.1 (23.6, 28)	25.6 (23.7, 28.8)	0.406 ^†^
ASA score				0.453 ^§^
1	1 (3.4%)	2 (11.1%)	3 (6.4%)	
2	20 (69.0%)	12 (66.7%)	32 (68.1%)	
3	8 (27.6%)	3 (16.7%)	11 (23.9%)	
Missing data	-	1 (5.6%)	1 (2.1%)	
Comorbidities				
Coronary artery disease	0 (0%)	3 (16.7%)	3 (6.4%)	0.023 ^*§^
Diabetes mellitus	4 (13.8%)	2 (11.1%)	6 (12.8%)	0.789 ^§^
Renal insufficiency	3 (10.7%)	1 (5.6%)	4 (8.7%)	0.545 ^§^
Nicotine abuse	5 (17.2%)	3 (16.7%)	8 (17.0%)	0.959 ^§^
Anemia	16 (55.2%)	7 (38.9%)	23 (48.9%)	0.227 ^§^
Missing data	1 (3.4%)	-	1 (2.1%)	
Height of tumor				0.406 ^§^
Upper third of rectum	7 (24.1%)	7 (38.9%)	14 (29.8%)	
Middle third of rectum	15 (51.7%)	9 (50.0%)	24 (51.1%)	
Lower third of rectum	7 (24.1%)	2 (11.1%)	9 (19.1%)	
Clinical T category				0.131 ^§^
T1	1 (3.4%)	1 (5.6%)	2 (4.3%)	
T2	4 (13.8%)	7 (38.9%)	11 (23.4%)	
T3	13 (44.8%)	5 (27.8%)	18 (38.3%)	
T4	7 (24.1%)	2 (11.1%)	9 (19.1%)	
Missing data	4 (13.8%)	4 (22.2%)	7 (14.9%)	
Clinical N category				0.004 ^*§^
N0	2 (6.9%)	10 (55.6%)	12 (25.5%)	
N1	2 (6.9%)	0 (0%)	2 (4.3%)	
N2	4 (13.8%)	0 (0%)	4 (8.6%)	
Nx	2 (6.9%)	0 (0%)	2 (4.3%)	
N+	12 (41.4%)	5 (27.8%)	17 (36.2%)	
Missing data	7 (24.1%)	3 (16.7%)	10 (21.3%)	
	**NT (*n* = 29)**	**No NT (*n* = 18)**	**Total (*n* = 47)**	***p*-Value**
mrCRM				0.109 ^§^
Positive	8 (27.6%)	2 (11.1%)	10 (21.3%)	
Uncertainly negative	1 (3.4%)	0 (0%)	1 (2.1%)	
Negative	7 (24.1%)	11 (61.1%)	18 (38.3%)	
Missing data	13 (44.8%)	5 (27.8%)	18 (38.3%)	
Pathological T category				0.391 ^§^
T0	4 (13.8%)	2 (11.1%)	6 (12.8%)	
Tis	0 (0%)	1 (5.6%)	1 (2.1%)	
T1	0 (0%)	1 (5.6%)	1 (2.1%)	
T2	5 (17.2%)	5 (27.8%)	10 (21.3%)	
T3	16 (55.2%)	6 (33.3%)	22 (46.8%)	
T4	2 (6.9%)	2 (11.1%)	4 (8.6%)	
Missing data	2 (6.9%)	1 (5.6%)	3 (6.4%)	
Pathological N category				0.613 ^§^
N0	17 (58.6%)	13 (72.2%)	30 (63.8%)	
N1	6 (20.7%)	2 (11.1%)	8 (17.0%)	
N2	4 (13.8%)	2 (11.1%)	6 (12.8%)	
Missing data	2 (6.9%)	1 (5.6%)	3 (6.4%)	
pCRM				0.298 ^§^
Positive	2 (6.9%)	0 (0%)	2 (4.3%)	
Negative	21 (72.4%)	18 (100%)	39 (83.0%)	
Missing data	6 (20.7%)	0 (0%)	6 (12.7%)	
Performed surgery				0.278 ^§^
Anterior resection	3 (10.3%)	2 (11.1%)	5 (10.6%)	
Low anterior resection	23 (79.3%)	14 (77.8%)	37 (78.7%)	
Intersphincteric resection	2 (6.9%)	0 (0%)	2 (4.3%)	
Proctocolectomy	1 (3.4%)	2 (11.1%)	3 (6.3%)	
Surgical approach of operation				0.100 ^§^
Robotic assisted	14 (48.3%)	3 (16.7%)	17 (36.2%)	
Laparoscopically assisted	6 (20.7%)	9 (50%)	15 (31.9%)	
Primarily open	6 (20.7%)	3 (16.7%)	9 (19.1%)	
Conversion from laparoscopy to open	2 (6.9%)	3 (16.7%)	5 (10.6%)	
Conversion from robotic to open	1 (3.4%)	0 (0%)	1 (2.1%)	
Creation of protective ileostomy	27 (93.1%)	18 (100%)	46 (95.7%)	0.255 §

Data were *n* (%) or median (25%, 75% interquartile range); ^§^ chi-square test; * *p* ≤ 0.05; ^†^ Mann–Whitney-U test; NT neoadjuvant therapy including radio-chemotherapy or exclusively radiotherapy or chemotherapy; BMI body mass index; normal weight BMI 18.5–25 kg/m^2^; overweight BMI > 25 kg/m^2^; ASA American Society of Anesthesiology; renal insufficiency creatinine >0.9 mg/dL in females and >1.2 mg/dL in males; anemia hemoglobin <12.0 g/dL in females and <13.5 g/dL in males; height of tumor upper third of rectum 12–16 cm from the anal verge, middle third of rectum 6–12 cm from the anal verge, lower third of rectum 0–6 cm from the anal verge; T tumor; Tis carcinoma in situ, T1 limited to mucosa and submucosa, T2 extension into but not through muscularis propria, T3 invasion of perirectal fat, T4 invasion of adjacent structures; N lymph nodes; N0 no involved lymph nodes, N1 metastasis in one to three perirectal nodes, N2 metastasis in four to more perirectal nodes, Nx regional lymph nodes cannot be assessed, N+ regional lymph nodes may be involved; mrCRM circumferential resection margin in pretherapeutic pelvic MRI; pCRM circumferential resection margin on pathology result.

**Table 2:** Different regimes of neoadjuvant therapy in patients with endoscopic vacuum therapy.

Regime	Patients (*n* = 29)
Short-term radiotherapy (5 × 5 gray)	3 (10.3%)
Long-term radiotherapy (six weeks, 50.4 gray)	6 (20.7%)
Total neoadjuvant therapy (long-term radiotherapy with capecitabine and leucovorin, fluorouracil, irinotecan and oxaliplatin)	2 (6.9%)
Long-term radiotherapy and chemotherapy (capecitabine)	9 (31.0%)
Long-term radiotherapy and chemotherapy (fluorouracil)	4 (13.8%)
Long-term radiotherapy and other chemotherapy (cisplatin, oxaliplatin)	2 (6.9%)
Chemotherapy	1 (3.4%)
No information on the regime of neoadjuvant therapy	2 (6.9%)

Data were *n* (%).

**Table 3:** Surgical characteristics of patients with endoscopic vacuum therapy with and without neoadjuvant therapy.

	NT (*n* = 29)	No NT (*n* = 18)	Total (*n* = 47)	*p*-Value
Technique of anastomosis				0.880 ^§^
Circular stapler	22 (75.9%)	14 (77.8%)	36 (76.6%)	
Hand-sewn	7 (24.1%)	4 (22.2%)	11 (23.4%)	
Technique of anastomosis				0.127 ^§^
Coloplasty pouch	4 (15.4%)	0 (0%)	4 (9.3%)	
End-to-end anastomosis	17 (65.4%)	13 (76.5%)	30 (69.8%)	
Side-to-end anastomosis	4 (15.4%)	1 (5.9%)	5 (11.6%)	
Ileum-J-pouch	1 (3.8%)	3 (17.6%)	4 (9.3%)	
Duration of surgery (minutes)	370 (284.5, 418)	326 (263, 407.3)	354 (276, 411)	0.309 ^†^
Time between operation and onset of anastomotic leakage	8 (6, 13.8)	8 (6, 14.3)	8 (6, 13.3)	0.717 ^†^
Site of anastomotic leakage				0.177 ^§^
Dorsal	9 (31.0%)	6 (33.3%)	15 (31.9%)	
Ventral	4 (13.8%)	1 (5.6%)	5 (10.6%)	
Right lateral	8 (27.6%)	2 (11.1%)	10 (21.3%)	
Left lateral	2 (6.9%)	6 (33.3%)	8 (17.0%)	
Circumferential	4 (13.8%)	1 (5.6%)	5 (10.6%)	
No information	2 (6.9%)	2 (11.1%)	4 (8.5%)	
Size of cave of insufficiency in rectoscopy (in millimeter) Missing data = 32	80 (52.5, 80)	40 (30, 80)	60 (40, 80)	0.111 ^†^

Data were *n* (%) or median (25%, 75% interquartile range); NT neoadjuvant radio-chemotherapy or exclusively radiotherapy or chemotherapy; EVT endoscopic vacuum therapy; ^§^ chi-square test; ^†^ Mann–Whitney-U test.

**Table 4:** Course of endoscopic vacuum therapy with and without neoadjuvant therapy.

	NT (*n* = 29)	No NT (*n* = 18)	Total (*n* = 47)	*p*-Value
Number of changes in sponge of EVT	5 (2, 13.5)	5 (2.8, 7.3)	5 (2, 8.3)	0.786 ^†^
Persistence of anastomotic leakage during EVT	6 (20.7%)	3 (16.7%)	9 (19.1%)	0.733 ^§^
Dissolving of anastomosis and creation of colostomy	4 (13.8%)	1 (5.6%)	5 (10.6%)	0.343 ^§^
De novo endoscopic vacuum therapy	1 (3.4%)	2 (11.1%)	3 (6.4%)	0.134 ^§^
Re-laparotomy and creation of jejunostomy	1 (3.4%)	0 (0%)	1 (2.1%)	-
Late onset recurrence of anastomotic leakage after initial healing	2 (6.9%)	2 (11.1%)	4 (8.5%)	0.615 ^§^
Long-term treatment failure of EVT	8 (27.6%)	5 (27.8%)	13 (27.7%)	0.989 ^§^
Length of hospital stay (days)	24 (13, 35)	25 (20.3, 30.3)	24 (14, 33)	0.606 ^†^
Adjuvant chemotherapy	5 (17.2%)	2 (11.1%)	7 (14.9%)	0.552 ^§^
Missing data	7 (24.1%)	3 (16.7%)	10 (21.3%)	
Reversal of ostomy	19 (79.2%)	11 (68.8%)	30 (75.0%)	0.456 ^§^
Missing data = 6				
No initial ileostomy = 1				
Anastomotic stricture	4 (13.8%)	5 (27.8%)	9 (19.1%)	0.236 ^§^

Data were *n* (%) or median (25%, 75% interquartile range); NT neoadjuvant radio-chemotherapy or exclusively radiotherapy or chemotherapy; EVT endoscopic vacuum therapy; ^§^ chi-square test; ^†^ Mann–Whitney-U test; long-term treatment failure of EVT included early persistence of anastomotic leakage during EVT and late onset of recurrence of anastomotic leakage.

## Data Availability

The raw data supporting the conclusions of this article will be made available by the authors on request.

## Abbreviations

ASA	American Society of Anesthesiology
BMI	body mass index
EVT	endoscopic vacuum therapy
nT	neoadjuvant therapy
TME	total mesorectal excision
UICC	union for international cancer control
